# Molecular Resiliency and Aging

**DOI:** 10.1093/geroni/igab046.619

**Published:** 2021-12-17

**Authors:** Adam Salmon

## Abstract

Resilience is described as the ability to respond to acute forms of stress and recover to normal homeostasis. There is growing evidence that biology of resilience is entwined with the biology of aging. With increasing age, resilience decreases and is a likely contributor to increased morbidity, frailty and susceptibility to death with age. Conversely, increased resilience across numerous physiological markers of function is associated with longevity and healthy aging. The variation in resilience in populations suggests biological and molecular regulatory mechanisms that might provide insight into interventions to improve resilience, healthy aging and longevity. In this session, speakers will provide insight regarding short-term assays of resilience in animal models that prove useful both in delineating these biological mechanisms as well as inform on potential translational models to better understand biological resilience in human populations. The sessions focus is on defining these assays and discussion of the biological relevance each resilience assay in terms of the regulation of aging. The goals of these studies range from identifying potential predictors of individual lifespan within markers of functional resilience to leveraging geroscience to define whether markers of resilience can be modified through interventions to the aging process. Moreover, better understanding of the biology of resilience could assist in defining novel interventions that improve resilience and thereby enhance longevity.

